# Limbal Squamous Cell Carcinoma in a Black Baldy Cow: Case Report and Surgical Treatment

**DOI:** 10.1155/2023/2429241

**Published:** 2023-02-15

**Authors:** Alexandra T. J. Ng, Richard J. McMullen, Gillian C. Shaw, Thomas Passler, Jenna Stockler

**Affiliations:** ^1^Auburn University College of Veterinary Medicine, JT Vaughan Large Animal Teaching Hospital, Auburn, AL, USA; ^2^Comparative Ocular Pathology Laboratory of Wisconsin, Department of Pathobiological Sciences, School of Veterinary Medicine, University of Wisconsin, Madison, WI, USA

## Abstract

**Objective:**

To document a case of limbal squamous cell carcinoma (SCC) in an adult Black Baldy cow treated with photodynamic therapy (PDT) as an adjunctive therapy following surgical excision. *Animals Studied*. One privately owned 8-year-old female, entire, Black Baldy cow. *Procedures*. A complete ophthalmic examination was performed on an adult Black Baldy cow for assessment of a mass affecting the left eye. Following a routine partial incision superficial lamellar keratectomy and conjunctivectomy under local analgesia using a Peterson retrobulbar block, photodynamic therapy was performed as an adjunctive treatment to lower the chance for recurrence and improve the prognosis for the globe.

**Results:**

Histopathologic analysis of the limbal mass was reported to be consistent with a squamous cell carcinoma, removed with clean margins. The patient was comfortable and visual with no signs of tumor recurrence 11 months after surgery.

**Conclusion:**

Superficial lamellar keratectomy and conjunctivectomy with adjunctive photodynamic therapy is an effective treatment for limbal squamous cell carcinoma and may be performed as an alternative to enucleation, exenteration, euthanasia, or slaughtering in cattle.

## 1. Introduction

Ocular squamous cell carcinoma (OSCC) is a common tumor of the eye in cattle. It most commonly affects the corneoscleral junction but can also affect the conjunctiva and ocular adnexa [1, 2].

Several contributing factors have been suggested for the development of OSCC including age, gender, breed, periocular and corneoscleral pigmentation, exposure to sunlight, viral infection, and nutrition. Predisposition of the Hereford breed and Hereford crosses has been well established; however, cases have been reported in many other breeds including Holstein-Friesian, Guernsey, Shorthorn, Ayrshire, Brahman, Brown Swiss, Mongolian, Jersey, and Normandy [3–5]. Initially, bovine OSCC can appear as a preneoplastic, raised, white plaque or frond-like papilloma on the epithelium around the eye that can progress to a malignant stage, characterized by a more irregular, pink, vascular nodule.

Limbal and corneal squamous cell carcinomas may respond to excision via keratectomy, often with adjunctive therapy including cryotherapy, hyperthermia, radiation therapy, carbon dioxide laser ablation, immunotherapy, or a combination of these therapies. If extension into orbit and ocular adnexa exists, enucleation or exenteration is recommended. OSCC in cattle has a significant economic impact, often leading to euthanasia or slaughtering of the animal [6, 7]. It has been reported that 12.6-82% of euthanasia or slaughtering for neoplasia at slaughter were due to OSCC in the United States [2, 4, 8].

Photodynamic therapy (PDT) is an adjunctive therapy that involves injecting infracyanine green, a photosensitive dye, directly onto the affected area and activating this dye with an infrared laser. The mechanism of action involves the production of oxygen radicals which causes the destruction of the neoplastic cells. The use of PDT has been described in human, canine, feline, and equine SCC with promising outcomes [9–13]. Photothermal therapy (PTT) is a similar technique that involves near-infrared light, nanoparticles, and a photothermal agent. When this agent is activated by a light of a suitable wavelength, heat is generated to destroy neoplastic cells [14–16]. Phototherapy techniques (PDT and PTT) have several advantages compared to other therapies, including minimally invasive procedures, less trauma to surrounding tissues, and reduced toxicity.

This report describes the use of infracyanine (EmunDo®, Richmond, BC, Canada) green-based photodynamic/photothermal therapy following surgical excision of an uncommon presentation of limbal (ocular) squamous cell carcinoma under local analgesia in a Black Baldy cow.

## 2. Case Report

### 2.1. Patient Information

A privately owned 8-year-old, female, entire Black Baldy cow was presented to Auburn University JT Vaughan Large Animal Teaching Hospital for evaluation of a white, proliferative mass on the left cornea. The mass had been present for approximately 2 weeks as reported by the owner.

### 2.2. Clinical Findings

**Figure 1:**
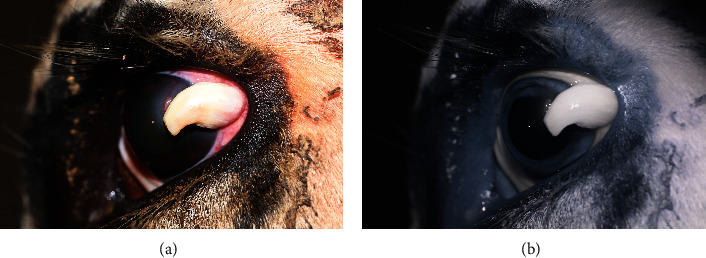
Color (a) and infrared (b) imaging of the limbal mass on initial examination of the left eye.

On presentation, the patient was bright, alert, and responsive with a normal mentation. Rectal palpation confirmed a 45-day pregnancy. A complete ophthalmic examination was performed and included slit-lamp biomicroscopy (SL-17, Kowa, Tokyo, Japan), Schirmer tear test, rebound tonometry (Icare® TONOVET tonometer, Vantaa, Finland), and indirect ophthalmoscopy. The cow was visual with a positive menace response, dazzle reflex, and palpebral reflex in both eyes (OU). Direct and consensual pupillary light reflexes were intact OU. Schirmer tear test results were 28 mm/60 seconds in the right eye (OD) and 27 mm/45 seconds in the left eye (OS). Intraocular pressure was estimated via rebound tonometry and was 27 mmHg OD and 20 mmHg OS. Ocular abnormalities OD included faint, focal, axial, anterior stromal fibrosis. Abnormal ocular findings in OS included a raised, white, exophytic mass originating from the temporal limbus extending to the medial paraxial cornea (approximately 20 mm long × 10 mm wide × 8 − 10 mm thick) with perilesional corneal edema 1-2 mm beyond the leading edge of the base of the mass (Figure 1).

Based on the ophthalmic examination, a working clinical diagnosis of suspected limbal squamous cell carcinoma OS was made. Differential diagnoses included papilloma, lymphoma, parasitic infestation, and inflammatory granuloma. Based on the clinical suspicion for limbal SCC, surgical excision with adjunctive photodynamic therapy (PDT) and histopathologic evaluation of the excised tissue were recommended and pursued.

### 2.3. Therapeutic Intervention

Following routine sterile preparation (dilute baby shampoo, 1% betadine solution, and sterile saline rinse), a Peterson retrobulbar block [17] was performed. Briefly, the notch bordered by the supraorbital process, zygomatic arch, and coronoid process was located, and a slightly curved 15 cm, 18-gauge spinal needle was inserted and directed rostromedially towards the pterygopalatine fossa. The spinal needle was advanced until the bony plate near the pterygopalatine fossa was reached (approximately 8 cm depth). The needle was then pulled back approximately 1.5 cm from the bone. Aspiration ensured that the ventral maxillary artery had not been penetrated. Ten milliliters (ml) of mepivacaine were then injected. The needle was then redirected ventrorostrally where an additional 10 ml of mepivacaine was deposited. The needle was retracted as the last of the 10 ml of mepivacaine was being injected.

**Figure 2:**
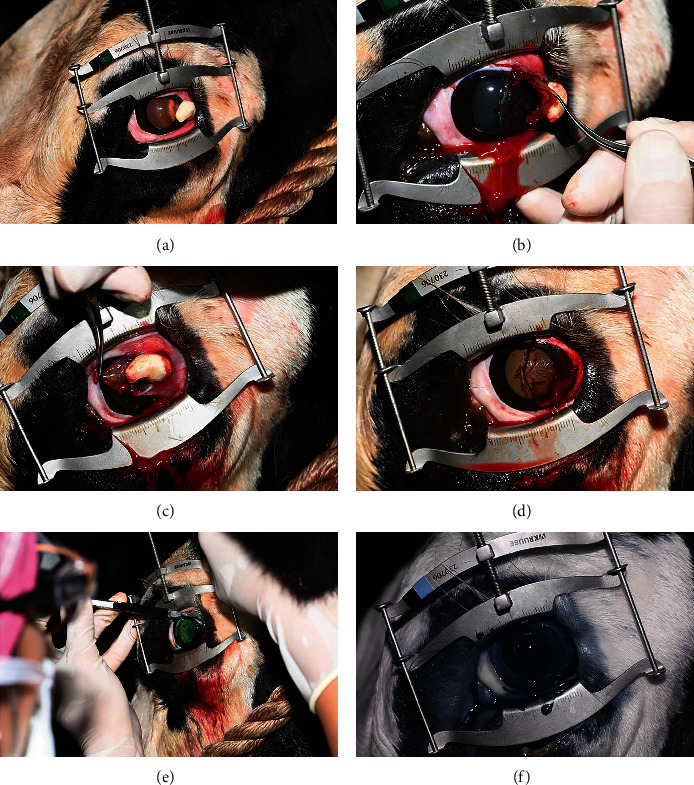
A partial incision superficial lamellar keratectomy (PI-SLK) and conjunctivectomy; removal of the corneoconjunctival section (a–d), “painting” of infracyanine green onto the keratectomy site and injection into the perilimbal conjunctiva (e), and infrared imaging showing no gross evidence of infiltrate (f).

A partial incision superficial lamellar keratectomy (PI-SLK) [18, 19] and conjunctivectomy was performed (Figures 2(a)–2(d)). An initial vertical, linear (4 mm length) corneal groove incision was performed in the axial cornea to approximately 30% stromal depth using a 6400 microsurgical blade. A Martinez corneal dissector was then inserted through the initial corneal groove incision, and the temporal half of the superficial corneal stroma was completely undermined to the limbus, using small circular advancing motions. The peripheral incision was extended to the dorsal and ventral limbus using curved corneal section scissors. The incisions were extended across the limbus with Westcott tenotomy scissors, which were then used to transect the limbus from the 1 to 6 o'clock positions. The corneoconjunctival section was then removed using 0.12 mm Colibri forceps and placed in formalin. Epinephrine and cotton absorbent sticks were used throughout the procedure to achieve hemostasis. Epinephrine was inadvertently injected subconjunctivally which resulted in only minimal local chemosis at the injection site.

Following PI-SLK and conjunctivectomy, photodynamic therapy was performed as follows: one milliliter of EmunDo® (infracyanine green (InfraCG)) was applied topically (“painted”) to the keratectomy site and injected into the perilimbal conjunctiva until the entire surgical site was coated with InfraCG (Figure 2(e)). The cornea was then irradiated with diffuse infrared laser energy using a FOX A.R.C. diode laser emitting light with a wavelength of 810 nanometer using a special light handpiece affixed to the tip of a dual-sided attachment fiber. Power output was maintained at 500 milliwatt (mW), with the probe being placed approximately 10 mm away from the targeted corneal surface for treatment with the goal of applying 150 joules (J) of total energy to the surgical site via intermittent cycles 30 seconds (s) in duration (each 30 s cycle at 500 mW of energy = 15 J). The area of the cornea coated with InfraCG was repeatedly irradiated in 30 s cycles until a total of 167 J was delivered.

Postoperatively, the patient received a single injection of flunixin meglumine (Banamine, Merck Animal Health, Madison, NJ) (1.1 mg/kg) intravenously and was subsequently treated with oral meloxicam (Boehringer Ingelheim (Canada) Ltd., Burlington, Ontario) (1 mg/kg) once daily. Tulathromycin (Draxxin, Zoetis Inc. Kalamazoo, MI) (5 mg/kg) was administered once by subcutaneous route as a perioperative antibiotic.

### 2.4. Follow-Up and Outcomes

**Figure 3:**
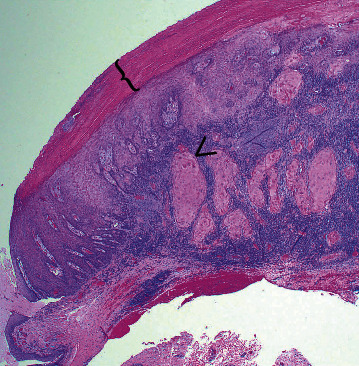
Lateral limbal lesion from a cow. Photomicrograph of the specimen showing the plaque-like exophytic neoplasm that replaced that conjunctival and corneal epithelium. The cells formed sheets and anastomosing lobules, which multifocally invade below the basement membrane (arrow). The superficial surface of the mass was composed of dense keratin (bracket). Inflammatory cells heavily infiltrated the underlying stroma. The margins were clear of neoplastic cells. Hematoxylin and eosin.

Histopathologic analysis of the mass revealed a squamous epithelial neoplasm that was focally invasive, directing the diagnosis of a squamous cell carcinoma. The lateral keratoconjunctivectomy specimen measured 22 × 16 × 12 mm with an exophytic firm off-white mass measuring 18 × 9 × 7 mm that spanned the limbus. Histologically, an unencapsulated, moderately well-demarcated, and densely cellular neoplasm replaced the epithelium at the limbus, formed an exophytic mass, and multifocally infiltrated into the superficial substantia propria where neoplastic cells were surrounded by plump spindle cells. The neoplastic cells formed sheets within large anastomosing lobules and trabeculae and were supported by a fine fibrovascular stroma. The cells were polygonal with variably distinct cell borders, moderate amounts to abundant finely fibrillar eosinophilic to amphophilic cytoplasm, and round to oval nuclei with finely dispersed to clumped chromatin and one to two prominent magenta nucleoli. More basally oriented cells were smaller with smaller amounts of more basophilic cytoplasm, and more superficial/centrally located cells were larger with abundant eosinophilic and occasionally glassy cytoplasm, typical of squamous differentiation. There was a very large proportion of keratinized cells in the exophytic portion of this neoplasm, some of which retained their nuclei. The centers of the invasive lobules were composed of large brightly eosinophilic cells, consistent with central keratinization. The cells exhibited marked anisocytosis and mild to moderate anisokaryosis. There were 28 mitotic figures in ten 400x fields (2.37 mm^2^) (Figure 3). The surrounding corneal and conjunctival epithelium was variably hyperplastic. Lymphocytes, plasma cells, and some polymorphonuclear cells multifocally occupied the surrounding stroma in varying numbers. Bacteria multifocally adhered to the surface of the mass (the keratinized cells). The sampled peripheral corneal stroma was vascularized. There was mild, multifocal substantia propria edema. The examined margins were clear of neoplastic cells. The deep margin was narrowest at approximately 250 *μ*m with loose conjunctival substantia propria comprising the marginal tissue.

**Figure 4:**
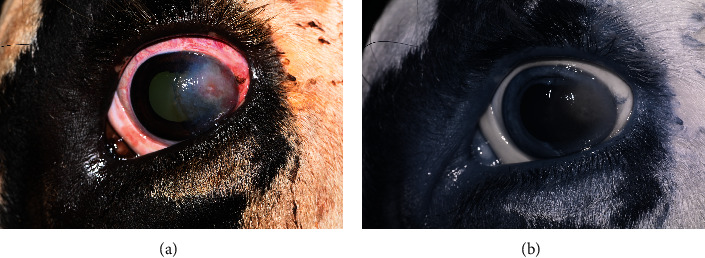
Color (a) and infrared (b) imaging of the left eye 1 week after surgery. Granulation of the keratectomy site is noted with no evidence of tumor regrowth.

**Figure 5:**
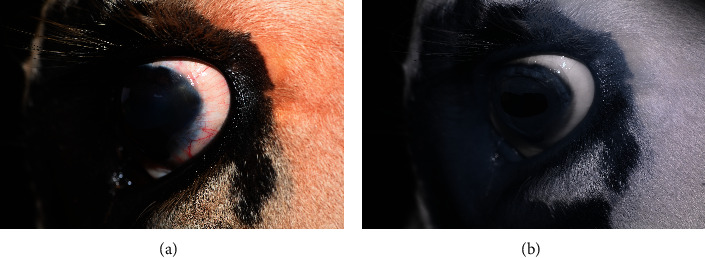
Color (a) and infrared (b) imaging of the left eye 11 months after surgery. Faint fibrosis is noted along the temporal limbus with no evidence of tumor regrowth.

The patient was evaluated 1 week after surgery and 11 months after surgery, at which time a limited ophthalmic examination was performed using color and infrared imaging. One week after surgery, the patient was comfortable with mild conjunctival hyperemia and granulation of the keratectomy site (Figure 4). Eleven months after surgery, the patient remained comfortable with only faint fibrosis of the cornea and no signs of tumor recurrence (Figure 5).

## 3. Discussion

This report documents a case of limbal squamous cell carcinoma in a Black Baldy cow, successfully removed by PI-SLK and subsequent adjunctive photodynamic therapy. Although ocular squamous cell carcinoma has been well characterized in cattle, this case report describes a novel adjunctive treatment option.

Several treatment options for SCC in various species include radiation therapy, immunotherapy, surgery, cryotherapy, and hyperthermia, with variable regression and recurrence rates [20]. Conclusions of these studies are often unreliable in cattle given the lack of published reports, follow-up, and confirmation of disease via histopathologic analysis. In addition, there is a lack of recent updated reports of SCC reported in cattle.

Local excision of small lesions of OSCC can be curative [20]; however, recurrence of tumor growth is common following surgical excision alone. Recurrence rate for equine SCC with surgery alone is higher compared to surgery with adjunctive radiation therapy [21, 22], with a mean tumor recurrence reported to be 449 ± 339 days [22]. Successful treatment may also become more challenging following tumor recurrence after an unsuccessful initial treatment [12]. One study reported regrowth rates between 4.4 and 13.3% after cryotherapy alone of lesions in Hereford cattle; however, confirmation of the lesions via histopathology was not performed for all lesions in this study, and time to recurrence was not reported [23]. Treatment using immunotherapy with bacillus Calmette-Guerin (BCG) has shown recurrence rates between 0 and 50%, with local recurrence occurring between 3 and 18 months after initial regression [24, 25]. The use of immunotherapeutic, chemotherapeutic, or radioactive agents requires special handling protocols and isolation for the animal and may result in expensive, or even illegal, outcomes in food-producing animals. Significant side effects including swelling, discomfort, healthy tissue damage, and keratitis also commonly occur.

This case report showed no evidence of tumor recurrence 11 months after surgical resection with PDT. Photodynamic therapy has no known toxic risks associated with handling and does not require the animal be placed in isolation. As infracyanine green is poorly absorbed systemically with ophthalmic administration, residues should not be of concern. Indocyanine green, a similar compound, has been used for intrastromal injections in horses and demonstrated green discoloration for 3 months. In humans, this compound has a 3-30 minute half-life following intravenous injection. For infracyanine green use in cattle, a conservation 105-day meat withdrawal should be considered (Personal communication – US Food Animal Residue Avoidance Databank http://www.farad.org). Additionally, PDT does not require general anesthesia and may be readily performed in a standing large animal patient under mechanical and/or manual restraint and local anesthesia with or without sedation. Along with minimal damage to the surrounding tissue, animals receiving PDT remain comfortable postoperatively [12, 13]. Horses with periocular SCC treated with surgical resection and PDT using verteporfin remained disease-free for a minimum of 25 months, while those treated with surgical excision and cryotherapy yielded a median time to tumor recurrence of 10 months [12, 13]. Disadvantages of PDT include the initial cost of the diode laser and availability of specific photosensitive agents. As stated in previous reports, early diagnosis and intervention of OSCC is essential to allow for successful treatment and prevent local invasion that would require enucleation, exenteration, or euthanasia or slaughtering. Currently unpublished data in the horses treated for OSCC suggests that InfraCG-based PDT may only require a single treatment and that recurrences are uncommon (Personal communication – McMullen).

In conclusion, to the authors' knowledge, this is the first report of photodynamic therapy after PI-SLK for OSCC in cattle. This surgical and therapeutic approach is a safe and effective treatment for limbal squamous cell carcinoma and may be performed as an alternative to enucleation, exenteration, euthanasia, or slaughter in cattle. Further studies are needed to determine recurrence of limbal SCC after surgical excision and PDT in cattle.
